# Establishment of efficient regeneration protocol for three rapeseed cultivars

**DOI:** 10.1080/13102818.2014.901668

**Published:** 2014-06-04

**Authors:** Emine Selcen Darçın, Özer Kolsarıcı, Mustafa Yıldız

**Affiliations:** ^a^ Department of Field Crops, Faculty of Agricultural Science and Technologies, Bilecik Şeyh Edebali University, Gülümbe, Bilecik, Turkey; ^b^ Department of Field Crops, Faculty of Agriculture,University of Ankara, Ankara, Turkey

**Keywords:** disinfectant, rapeseed, root formation, seedling growth, shoot regeneration

## Abstract

Economically, rapeseed is one of the most important crops in the world. Over the past decades, rapeseed research has been focused on improving biotechnological methods to facilitate breeding. The effectiveness of these methods depends on efficient tissue culture *techniques.* The aim of the present study was to establish an efficient protocol for regeneration of rapeseed. It was conducted in three stages. The first stage was to determine the effect of different concentrations (10%, 20%, 30% and 40%) of sodium hypochlorite solutions on seedling growth parameters. In the second stage, the effects of different growth media (Murashige and Skoog, MS, and Gamborg) and plant growth regulators (6-benzylaminopurine, 1-naphthaleneacetic acid and thidiazuron) at different concentrations on the regeneration capacity of stem explants of three rapeseed cultivars were investigated. In the last stage, the shoots of ‘Spok’ were cultured for three weeks on MS medium containing 1.5, 3 and 4.5 mg L^−1^ of indol butyric acid for rooting. The best results in germination, seedling growth and root length were obtained by using 10% disinfectant concentration for 25 minutes. The highest values for shoot regeneration were obtained from the stem explant cultured on MS medium containing 3 mg L^−1^ BAP and 0.2 mg L^−1^ NAA. It was found that MS containing 1.5 mg L^−1^ IBA was the most efficient medium for root formation.

## Abbreviations

B5: Gamborg medium
BAP: 6-benzylaminopurine
IBA: indol butyric acid
MS: Murashige and Skoog medium
NAA: 1-naphthaleneacetic acid
TDZ: thidiazuron

## Introduction

Rapeseed (*Brassica napus* ssp. *oleifera* L.), *Crucifera* family, is widely used as an oil crop worldwide. It is also used as protein-rich livestock feed, as a source of biodiesel, lamp oil, soap, and plastics, as an excellent rotating crop for soil management, an effective heavy metal hyperaccumulator plant, a medicinal plant with a diuretic, anti-scurvy and anti-inflammatory (bladder) and anti-gout effect in Middle Asia, North Africa and West Europe.[1]

Over the past decades, rapeseed research has been focused on improving biotechnological methods to facilitate breeding. The application of biotechnology to crop improvement requires efficient regeneration protocols. The adventitious shoot regeneration capacity of plant cells or tissues is crucial in transformation techniques applied for introduction of foreign genes coding for agronomically important characters.[2,3] In addition, factors such as age and type of explant, genotype, growth medium, phytohormones, additives, disinfectant, and sources of carbon determine the success of plant regeneration.[4,5]

Surface sterilization is the first step in all tissue culture studies. Ideally, its purpose is to eliminate all microorganisms that can easily grow *in vitro*, without damaging the tissue's viability and regeneration capacity. Explant regeneration capacity is known to be affected by temperature,[6] concentration and disinfectant application time.[7]

The aim of this study was to determine the effects of sodium hypochlorite solutions at different concentrations on *in vitro* seed germination and seedling growth. The effects of different growth media (MS and B5) and plant growth regulators (BAP, NAA and TDZ) at different concentrations and combinations on the regeneration capacity of hypocotyl and stem explants of three rapeseed cultivars (‘Westar’, ‘Helios’ and ‘Spok’) were also analysed.

## Materials and methods

Seeds of *Brassica napus* ssp. *oleifera* L. cvs. ‘Spok’, ‘Helios’ and ‘Westar’ were surface sterilized with commercial bleach (containing 5% sodium hypochlorite) at four concentrations (10%, 20%, 30% and 40%) for 25 minutes with continuous stirring and then they were rinsed three times with sterile water. Percentage seed germination, seedling growth and root length were observed seven days after culture initiation.

Sterilized seeds were germinated on a basal medium containing the mineral salts and vitamins of Murashige and Skoog (MS),[8] 3% (w/v) sucrose and 0.7% (w/v) agar. All cultures were incubated at 25 °C under cool white fluorescent light (27 μmol m^−2^ s^−1^) with a 16 h light/8 h dark photoperiod. The pH of the medium was adjusted to 5.8 prior to autoclaving.

In order to determine the shoot regeneration capacity, hypocotyl segments, 5 mm in length, were excised from seven-day-old seedlings and stem segments, 3–4 mm in length, were excised from 3 to 4 week old seedlings obtained from the seeds that were sterilized with 10% disinfectant concentration for 25 minutes. All explants were cultured on MS and B5 [9] with different combinations of 6-benzylaminopurine (BAP), 1-naphthaleneacetic acid (NAA) and thidiazuron (TDZ). Shoot regeneration percentage, shoot number per hypocotyl, shoot length and total shoot number per Petri dish were recorded six weeks after culture initiation. The same parameters per stem were scored four weeks after the beginning of the experiment.

The shoots of ‘Spok’ were cultured for three weeks on MS medium containing 1.5, 3 and 4.5 mg L^−1^ indol butyric acid (IBA) for rooting. Rooted shoots were transferred to pots containing soil for acclimation and cultured in an acclimatization room under a 16 h light/8 h dark photoperiod at 20 °C–23 °C with relative humidity (100%) for three weeks.

Three replicates were tested and there were 10 explants per replication. Data were statistically analysed by using Duncan's multiple range test (SPSS for Windows 11.00 program). Data given in percentages were subjected to arcsine (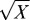) transformation before statistical analysis.[10]

## Results and discussion

### Effect of disinfectant concentrations

According to the results, the overall scores of seedling length (*P* < 0.05), germination percentage and root length (*P* < 0.01) were statistically significant (Table 1). The germination percentage varied from 77.33% to 0.00% for ‘Spok’, from 99.17% to 0.00% for ‘Westar’ and from 92.67% to 20.33% for ‘Helios’. Similar results were observed for the other parameters studied. There were significant declines in all parameters of the three rapeseed cultivars at 40% concentration of disinfectant (Table 1). The highest results in all parameters were obtained when surface sterilization was performed with 10% disinfectant concentration for 25 minutes. Lower values were recorded in seed germination percentage, seedling and root lengths by increasing concentrations of sodium hypochlorite solutions.

**Table 1.** Effect of NaOCl concentrations on *in vitro* seed germination and seedling growth of rapeseed cultivars.

		Disinfectant concentration (%)			
Cultivars	Parameters	10	20	30	40
‘Spok’	Seed germination (%)	77.33^a^	64.67^b^	20.67^c^	0.00^d^
	Seedling length (cm)	6.43^a^	4.57^a^	3.97^ab^	0.00^b^
	Root length (cm)	6.23^a^	3.29^b^	1.83^bc^	0.00^c^
‘Westar’	Seed germination (%)	99.17^a^	87.83^b^	66.50^c^	0.00^d^
	Seedling length (cm)	9.98^a^	8.48^a^	6.90^a^	0.00^b^
	Root length (cm)	6.91^a^	5.08^ab^	4.11^b^	0.00^c^
‘Helios’	Seed germination (%)	92.67^a^	76.67^b^	55.51^c^	20.33^d^
	Seedling length (cm)	8.23^a^	6.43^ab^	5.07^ab^	3.10^b^
	Root length (cm)	8.41^a^	5.40^b^	4.04^bc^	1.80^c^

Note: Values within a row for each cultivar followed by different letters are significantly different at the 0.01 level.

Surface sterilization of the explant is probably the most important step prior to culture initiation, since otherwise different microorganisms would find an optimal growth environment *in vitro*, which in turn would hinder the progress of the tissue culture study. Tissue sterilization, on one hand, aims to eliminate all bacteria and fungi that can easily grow *in vitro*, but on the other hand, it should also not harm the explant's viability and regeneration capacity which are known to be affected by the concentration, application period [7] and temperature [6] of disinfectant. Since direct contact of the explant with the disinfectant during the sterilization process can have a severe effect on regeneration frequency of the tissue,[6,11] it is highly recommended to use aseptic tissues as a source of explants.[12,13] A wide range of surface disinfectants, such as ethanol, hydrogen peroxide, bromine water, mercuric chloride, silver nitrate and antibiotics, are used for surface sterilization (reviewed in [26]); however, sodium hypochlorite (NaOCl) has been most widely used, as it is highly effective against all kinds of bacteria, fungi and viruses.[14–16]

It is well known that, the concentration, application period and temperature of NaOCl solutions used in the sterilization step in tissue culture experiments are closely related to each other and should be considered together. It has also been shown that the sterilization process not only affects seed germination and seedling growth directly, but also indirectly influences the regeneration capacity of explants and the health of the regenerated shoots.[6,11] Our results were are also in agreement with those of Allan [7] and Pierik,[17] who reported that the regeneration capacity of explants was negatively affected by higher concentrations and longer application periods of disinfectants.

### Shoot regeneration

Marked differences, which were all statistically significant at the 0.01 level, were observed in all parameters among the shoots regenerated from explants, medium and combinations of phytohormones.

**Figure 1.**
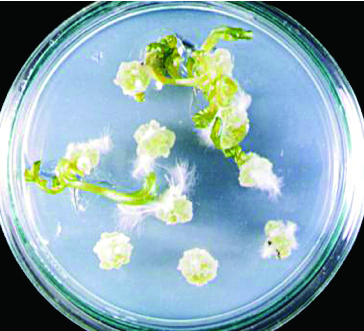
Shoot regeneration from hypocotyl explants of cv. ‘Spok’ on MS medium containing 3 mg L^−1^ BAP and 0.2 mg L^−1^ NAA.

**Figure 2.**
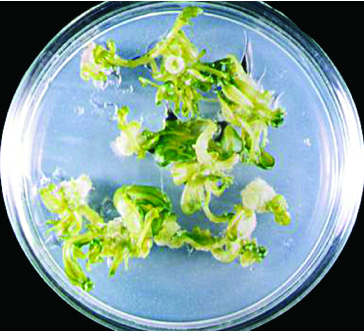
Shoot regeneration from stem explants of cv. ‘Spok’ on MS medium containing 3 mg L^−1^ BAP and 0.2 mg L^−1^ NAA.

First, different growth medium and phytohormone combinations in cv. ‘Spok’ stem and hypocotyl explants were applied (Table 2). It was observed that callus induction was 100%. The highest callus weight (0.626 g) and total shoot number per Petri dish (15.33) were obtained from hypocotyl explants cultured on MS medium containing 3 mg L^−1^ BAP + 0.2 mg L^−1^ NAA (Figure 1). The highest shoot regeneration (90%) was found to be in stem explants cultured on MS medium with 3 mg L^−1^ BAP + 0.2 mg L^−1^ NAA and 3 mg L^−1^ BAP + 0.4 mg L^−1^ NAA, respectively (Figure 2). Maximum shoot number per explant (12.97) and total shoot number per Petri dish (118) were obtained from stem explants cultured on MS medium with 3 mg L^−1^ BAP + 0.2 mg L^−1^ NAA (Table 1).

**Table 2.** *In vitro* shoot regeneration from hypocotyl and stem explants of cv. ‘Spok’ on different growth media containing different concentrations and combinations of BAP and NAA.

	Callus weight (g)		Shoot regeneration (%)		Shoot number per explant		Total shoot number per Petri dish	
Media	MS	B5	MS	B5	MS	B5	MS	B5
Hypocotyl								
1.5 mg L^−1^ BAP + 0.2 mg L^−1^ NAA	0.392^Ba1^	0.327^Aa1^	10.00 ^Aa1^	13.33^Aa1^	9.66^Aa1^	5.50^Ba1^	9.66^Ab1^	11.00^Aa1^
1.5 mg L^−1^ BAP + 0.4 mg L^−1^ NAA	0.408^Ba1^	0.251^Aa1^	16.66 ^Ab1^	10.00^Aa1^	4.00^Ab1^	13.33^ABa1^	8.00^Ab1^	13.00^Aa1^
3 mg L^−1^ BAP + 0.2 mg L^−1^ NAA	0.626^Aa1^	0.331^Aa2^	23.33^Ab1^	6.70^Aa1^	9.16^Aa1^	3.70^Ba1^	15.33^Aa1^	3.70^Ba1^
3 mg L^−1^ BAP + 0.4 mg L^−1^ NAA	0.612^Aa1^	0.534^Aa1^	20.00^Ab1^	0.00^Aa1^	3.11^Aa1^	0.00^Ba1^	8.00^Ab1^	0.00^Ba1^
Stem								
1.5 mg L^−1^ BAP + 0.2 mg L^−1^ NAA	0.359^Aa1^	0.083^Ab2^	33.33^Aa1^	10.00^a1^	4.97^Aa1^	4.00^Aa1^	19.33^Ba1^	5.00^Aa1^
1.5 mg L^−1^ BAP + 0.4 mg L^−1^ NAA	0.247^Ab1^	0.264^Ba1^	83.33^Ba1^	23.33^Aa1^	12.70^Aa1^	4.33^Aa1^	101.7^Aa1^	12.33^Aa2^
3 mg L^−1^ BAP + 0.2 mg L^−1^ NAA	0.383^Ab1^	0.197^Bb2^	90.00^Ba1^	36.70^Aa2^	12.97^Ba1^	10.20^Aa1^	118.0^Aa1^	34.30^Aa2^
3 mg L^−1^ BAP + 0.4 mg L^−1^ NAA	0.385^Ab1^	0.158^Bb2^	90.00^Ba1^	20.00^Aa2^	10.12^Aa1^	2.10^Aa1^	84.70^Aa1^	2.10^Aa2^

Note: Means followed by different capital letters for each growth medium and explant are significantly different at the 0.01 level. Means followed by different lower-case letters for each growth medium and hormone combination are significantly different at the 0.01 level. Means followed by different figures for each explant and hormone combination are significantly different at the 0.01 level.

In cv. ‘Westar’, it was observed that the highest callus induction was 94.56%. The highest callus weight (0.486 g), shoot regeneration (33.33%) and total shoot number per Petri dish (16.0) were recorded from stem explants cultured on MS medium with 3 mg L^−1^ BAP + 0.4 mg L^−1^ NAA (Table 3). On the other hand, the highest shoot number per explant (8.7) was observed with stem explants and regeneration medium with 1.5 mg L^−1^ BAP + 0.2 mg L^−1^ NAA.

**Table 3.** *In vitro* shoot regeneration from hypocotyl and stem explants of cv. ‘Westar’ on different growth media containing different concentrations and combinations of BAP and NAA.

	Callus weight(g)		Shoot regeneration (%)		Shoot number per explant		Total shoot number per Petri dish	
Media	MS	B5	MS	B5	MS	B5	MS	B5
Hypocotyl								
1.5 mg L^−1^ BAP + 0.2 mg L^−1^ NAA	0.352^Aa1^	0.295^ABa1^	6.70^Aa1^	0.00^Aa1^	2.00^Ab1^	0.00^Aa1^	2.00^Aa1^	0.00^Aa1^
1.5 mg L^−1^ BAP + 0.4 mg L^−1^ NAA	0.268^ABa1^	0.147^Ba1^	0.00^Aa1^	0.00^Aa1^	0.00^Aa1^	0.00^Aa1^	0.00^Aa1^	0.00^Aa1^
3 mg L^−1^ BAP + 0.2 mg L^−1^ NAA	0.393^Aa1^	0.238^ABa1^	3.33^Aa1^	3.33^Aa1^	2.33^Aa1^	2.00^Aa1^	2.33^Aa1^	2.00^Aa1^
3 mg L^−1^ BAP + 0.4 mg L^−1^ NAA	0.138^Bb2^	0.342^Aa1^	0.00^Ab1^	0.00^Aa1^	0.00^Ab1^	0.00^Aa1^	0.00^Ab1^	0.00^Aa1^
Stem								
1.5 mg L^−1^ BAP + 0.2 mg L^−1^ NAA	0.398^ABa1^	0.386^Aa1^	6.70^Aa1^	6.70^Aa1^	8.70^Aa1^	0.50^Aa2^	8.70^ABa1^	1.00^Aa1^
1.5 mg L^−1^ BAP + 0.4 mg L^−1^ NAA	0.242^Ba1^	0.254^Aa1^	0.00^Aa1^	6.70^Aa1^	0.00^Ca1^	0.70^Aa1^	0.00^Ba1^	0.70^Aa1^
3 mg L^−1^ BAP + 0.2 mg L^−1^ NAA	0.477^Aa1^	0.246^Aa2^	13.33^Aa1^	6.70^Aa1^	3.00^BCa1^	0.80^Aa1^	7.00^Aa1^	1.70^Aa1^
3 mg L^−1^ BAP + 0.4 mg L^−1^ NAA	0.486^Aa1^	0.336^Aa1^	33.33^Ba1^	6.70^Aa1^	6.70^ABa1^	1.00^Aa2^	16.00^Aa1^	2.00^Aa2^

Note: Means followed by different capital letters for each growth medium and explant are significantly different at the 0.01 level. Means followed by different lower-case letters for each growth medium and hormone combination are significantly different at the 0.01 level. Means followed by different figures for each explant and hormone combination are significantly different at the 0.01 level.

In cv. ‘Helios’, the mean callus induction was 99.8%. The highest callus weight (0.498 g) was observed in hypocotyl explants cultured on B5 medium containing 3 mg L^−1^ BAP + 0.2 mg L^−1^ NAA. Shoot regeneration (13.33%) was highest in B5 medium with 1.5 mg L^−1^ BAP + 0.4 mg L^−1^ NAA in hypocotyl explants (Table 4). The highest shoot number per explant (6.33) was obtained from stem explants cultured on 1.5 mg L^−1^ BAP + 0.4 mg L^−1^ NAA. On the other hand, the highest total shoot number per Petri dish was obtained from stem explant cultured on B5 medium with 3 mg L^−1^ BAP + 0.2 mg L^−1^ NAA (Table 4).

**Table 4.** *In vitro* shoot regeneration from hypocotyl and stem explants of cv. ‘Helios’ on different growth media containing different concentrations and combinations of BAP and NAA.

	Callus weight (g)		Shoot regeneration (%)		Shoot number per explant		Total shoot number per Petri dish	
	MS	B5	MS	B5	MS	B5	MS	B5
Hypocotyl								
1.5 mg L^−1^ BAP + 0.2 mg L^−1^ NAA	0.213^Ca2^	0.359^Ba1^	0.00^Aa1^	3.33^Aa1^	0.00^Aa1^	2.00^Aa1^	0.00^Aa1^	2.00^Aa1^
1.5 mg L^−1^ BAP + 0.4 mg L^−1^ NAA	0.254^Ca2^	0.448^ABa1^	0.00^Aa1^	13.33^Ba1^	0.00^Aa1^	3.20^Aa1^	0.00^Aa1^	3.20^Aa1^
3 mg L^−1^ BAP + 0.2 mg L^−1^ NAA	0.359^Ba2^	0.498^Aa1^	0.00^Aa1^	0.00^Aa1^	0.00^Aa1^	0.00^Aa1^	0.00^Aa1^	0.00^Aa1^
3 mg L^−1^ BAP + 0.4 mg L^−1^ NAA	0.463^Aa1^	0.307^ABa1^	0.00^Aa1^	0.00^Aa1^	0.00^Aa1^	0.00^Aa1^	0.00^Aa1^	0.00^Aa1^
Stem								
1.5 mg L^−1^ BAP + 0.2 mg L^−1^ NAA	0.260^Ba1^	0.307^Aa1^	0.00^Aa1^	0.00^Aa1^	0.00^Aa1^	0.00^Aa1^	0.00^Aa1^	0.00^Aa1^
1.5 mg L^−1^ BAP + 0.4 mg L^−1^ NAA	0.294^Ba1^	0.212^Ab1^	3.33^Aa1^	3.33^Aa1^	1.00^Aa1^	6.33^Ba1^	1.00^Aa1^	0.33^Aa1^
3 mg L^−1^ BAP + 0.2 mg L^−1^ NAA	0.381^ABa1^	0.220^Ab2^	6.70^Aa1^	6.70^Aa1^	0.83^Aa1^	7.33^Ba1^	1.70^Aa1^	7.33^Ba1^
3 mg L^−1^ BAP + 0.4 mg L^−1^ NAA	0.316^ABa1^	0.290^Aa1^	0.00^Aa1^	0.00^Aa1^	0.00^Aa1^	0.00^Aa1^	0.00^Aa1^	0.00^Aa1^

Note: Means followed by different capital letters for each growth medium and explant are significantly different at the 0.01 level. Means followed by different lower-case letters for each growth medium and hormone combination are significantly different at the 0.01 level. Means followed by different figures for each explant and hormone combination are significantly different at the 0.01 level.

Among the three cultivars, ‘Spok’ gave the best results. Differences among media, hormone combinations and explant types were also observed, and it was shown that shoot regeneration was clearly affected by them. The best results were achieved on MS medium containing 3 mg L^−1^ BAP + 0.2 mg L^−1^ NAA.

In our previous studies, stem explants of ‘Spok’ gave the best results. The highest values in callus weight (0.150 g), shoot regeneration (46.70%), shoot number per explant (10.7), total shoot number per Petri dish (46.33) were obtained from stem explants cultured on MS medium containing 0.15 mg L^−1^ TDZ + 3 mg L^−1^ BAP + 0.2 mg L^−1^ NAA (Table 5).

**Table 5.** Effect of different doses and combinations of TDZ+BAP+NAA on shoot regeneration from stem explants of cv. ‘Spok’.

TDZ (mg L^−1^)	BAP (mg L^−1^)	NAA (mg L^−1^)	Callus weight (g)	Shoot regeneration (%)	Shoot number per explant	Total shoot number per Petri dish
0.15	0	0	0.039^c^	6.70^d^	0.83^bc^	1.7^b^
0.30	0	0	0.026^c^	26.70^b^	2.10^bc^	7^b^
0.45	0	0	0.019^bc^	16.70^c^	1.44^bc^	4^b^
0.15	3	0.2	0.150^a^	46.70^a^	10.70^a^	46.33^a^
0.30	3	0.2	0.087^b^	26.70^b^	4.33^b^	12.7^b^
0.45	3	0.2	0.00^c^	0.00^e^	0.00^c^	0.00^b^

Note: Values within a column followed by different letters are significantly different at the 0.01 level.

NAA and BAP are commonly used as growth regulators in plant *in vitro* culture.[2,18,19] On the other hand, TDZ, a substituted phenylurea with a cytokinin-like effect,[20,21] has been widely used in *in vitro* culture studies recently. TDZ stimulates plant regeneration in some plant species via organogenesis or somatic embryogenesis [22] but at higher concentrations may have detrimental effects, e.g. necrosis in tissues,[23] hyperhydricity,[24] and abnormal leaf morphology.[25] The tolerable doses of TDZ differ depending on the genus and the type of explant used. For example, pea does not tolerate more than 2 mg L^−1^ TDZ,[26] while TDZ up to 30 mg L^−1^ results in high shoot regeneration in groundnut.[27]

High callus weight could be attributed to an increase in the absorption of water and other components from the basal medium, as highlighted by Yildiz and Özgen.[28] The increased shoot numbers per explant were reflected in higher values for total shoot number per Petri dish.

### Root formation

At the end of the study, the shoots of cv. ‘Spok’ were cultured on MS medium containing 1.5, 3.0 and 4.5 mg L^−1^ IBA for rooting. All shoots cultured on rooting medium formed roots. The highest root number per shoot (11.33) was obtained from MS medium containing 1.5 mg L^−1^ IBA, whereas the highest root length (3.70 cm) was observed on MS medium containing 4.5 mg L^−1^ IBA (Table 6).

**Table 6.** Effect of different IBA doses on root formation in cv. ‘Spok’.

IBA (mg L^−1^)	Root number per shoot	Root length (cm)
1.5	11.33^a^	1.98^b^
3.0	8.67^ab^	1.83^b^
4.5	7.00^b^	3.70^a^

Note: Values within a column followed by different letters are significantly different at the 0.01 level.

## Conclusions

In this study, the highest values in shoot regeneration were obtained from rapeseed stem explants cultured on MS medium containing 3 mg L^−1^ BAP and 0.2 mg L^−1^ NAA. MS containing 1.5 mg L^−1^ IBA was the most efficient medium for root formation. This efficient regeneration protocol could aid the application of transformation techniques for future crop improvement.
